# Significance of Kynurenine 3-Monooxygenase Expression in Colorectal Cancer

**DOI:** 10.3389/fonc.2021.620361

**Published:** 2021-04-16

**Authors:** Chun-Yu Liu, Tzu-Ting Huang, Ji-Lin Chen, Pei-Yi Chu, Chia-Han Lee, Hsin-Chen Lee, Yu-Hsuan Lee, Yuan-Ya Chang, Shung-Haur Yang, Jeng-Kai Jiang, Wei-Shone Chen, Yee Chao, Hao-Wei Teng

**Affiliations:** ^1^School of Medicine, National Yang-Ming University, Taipei, Taiwan; ^2^Division of Transfusion Medicine, Department of Medicine, Taipei Veterans General Hospital, Taipei, Taiwan; ^3^Division of Medical Oncology, Department of Oncology, Center for Immuno-Oncology, Taipei Veterans General Hospital, Taipei, Taiwan; ^4^School of Medicine, National Yang Ming Chiao Tung University, Hsinchu, Taiwan; ^5^Department of Pathology, Show Chwan Memorial Hospital, Changhua City, Taiwan; ^6^School of Medicine, Fu Jen Catholic University, New Taipei City, Taiwan; ^7^School of Medicine, Institute of Pharmacology, National Yang-Ming Chiao Tung University, New Taipei City, Taiwan; ^8^Division of Colon and Rectum Surgery, Department of Surgery, Taipei Veterans General Hospital, Taipei, Taiwan; ^9^Department of Surgery, National Yang Ming Chiao Tung University Hospital, Yilan, Taiwan

**Keywords:** kynurenine 3-monooxygenase, colorectal cancer, overall survival, metastasis, stemness

## Abstract

Colorectal cancer (CRC) is a leading cause of cancer-related deaths. Because of the lack of reliable prognostic and predictive biomarkers for CRC, most patients are often diagnosed at a late stage. The tryptophan–kynurenine pathway plays a crucial role in promoting cancer progression. Kynurenine is considered an oncometabolite in colon cancer, and its downstream metabolites are also associated with CRC. Kynurenine 3-monooxygenase (KMO), a pivotal enzyme that catalyzes kynurenine metabolism, is essential for several cellular processes. In the current study, we explored the role of KMO in CRC. Immunohistochemical results showed that KMO was upregulated in CRC tissues relative to paired healthy tissue and polyps. Moreover, CRC patients with higher KMO expression were associated with higher metastasis and poorer survival rates. Knockdown of KMO decreased the expression of cancer stem cell markers, as well as the sphere-forming, migration, and invasion abilities of CRC cells. Additionally, blockade of the enzymatic activity of KMO using an inhibitor suppressed sphere formation and cell motility in CRC cells. These findings suggest the clinical relevance of KMO in CRC tumorigenesis and aggressiveness.

## Introduction

Colorectal cancer (CRC) ranks as the third-highest cause of cancer-related deaths and has increasing incidence in Taiwan (1). Different molecular subtypes of CRC exhibit distinct genetic signatures and clinical outcomes. Mutations, including *RAS, BRAF, PIK3CA, APC, TP53* mutations, and loss of *PTEN* expression, are usually present in metastatic CRC, and some of these genes have been suggested as promising predictive markers, and some act as predictive markers (2, 3). Tumor metastasis, relapse, and drug resistance lead to poor prognosis in CRC, despite advances in CRC treatments, such as radiotherapy, surgery, and chemotherapy (4, 5). Considering the high morbidity and modest effectiveness of CRC treatment, identifying reliable biomarkers of prognosis and therapeutic targets for patients with CRC is of paramount importance.

Almost 95% of dietary tryptophan, an essential amino acid, is metabolized along the kynurenine pathway. The tryptophan–kynurenine pathway is a crucial mechanism in the control of epithelial–mesenchymal transition (EMT) and helps cancers escape immune surveillance (6–8). Tryptophan-2,3-dioxygenase (TDO) and indoleamine-2,3-dioxygenases (IDOs), the enzymes responsible for the first and rate-limiting steps of tryptophan catabolism to kynurenine, are crucial in limiting adaptive immune responses and are expressed in many malignant and inflammatory diseases (9). In the kynurenine pathway, kynurenine 3-monooxygenase (KMO), a flavoprotein hydroxylase located on the outer membrane of mitochondria, catalyzes the conversion of kynurenine to 3-hydroxykynurenine (3-HK) and is broadly expressed in various tissues and cell types (10). The metabolites of kynurenine play a crucial role in infection, inflammation, and maintenance of the immunosuppressive microenvironment in many types of cancers. Kynurenine metabolites promote CRC cell proliferation and inhibit cell apoptosis by activating the PI3K–Akt pathway (11). A higher 3-HK to 8-hydroxykynurenic acid ratio is associated with increased CRC risk (12). KMO serves as a therapeutic target in multiple-organ failure, systemic inflammatory response, Huntington's disease, and immune adaptive response (13, 14). Recently, upregulation of KMO in hepatocellular carcinoma and triple-negative breast cancer tissues has been reported (15, 16). These studies suggest that KMO participates in cancer progression, whereas the role of KMO in CRC tumorigenesis and aggressiveness has not yet been demonstrated. In this study, we characterized KMO as an oncogene and link it to poor outcomes in CRC.

## Materials and Methods

### Patient Specimens

A total of 242 medical samples from patients with CRC were obtained from the in-house Biobank of Taipei Veterans General Hospital (VGHTPE). This study was approved by the Institutional Review Board of Taipei Veterans General Hospital (IRB-TPEVGH) and conducted in compliance with the Helsinki Declaration. IRB-TPEVGH waived the requirement for informed consent. The clinicopathological stage was assessed based on the American Joint Committee on Cancer staging system, 7th edition. The clinical course was determined by searching a computer database containing detailed information. The experiments were performed in accordance with the approved guidelines and regulations.

### Immunohistochemical Staining and Histochemical Score Determination

The paraffin-embedded CRC tissue sections were deparaffinized with xylene for 5 min, followed by two changes of xylene; the slides were then rehydrated. Peroxidase activity was blocked using 3% H_2_O_2_ for 10 min. The slides were incubated with blocking solution (2% FBS and 1% bovine serum albumin) for 1 h at room temperature. Primary antibodies against KMO (Abcam, Cambridge, MA, USA) were used at 1:100 dilution for overnight incubation at 4°C. The slides were counterstained with hematoxylin stain solution followed by detection with an EnVision Detection Systems Peroxidase/DAB, Rabbit/Mouse kit (Agilent, Santa Clara, CA, USA) according to the manufacturer's instructions. MSI status was identified by mismatch repair protein expressions. Antibodies against MLH1 (clone M1), PMS2 (clone EPR3947), MSH2 (clone G219–1129), and MSH6 (clone 44) were used for immunohistochemical staining with a BenchMark ULTRA system (Ventana, Indianapolis, IN, USA) according to the manufacturers' recommendations. The protein levels were determined with a semiquantitative method represented as H-scores. The assessment of the H-scores was performed by a single medical oncologist (Dr. HW Teng) who was blinded to clinical information. The H-score (0–300) was determined using semiquantitative assessment and was calculated by multiplying the percentage of positively stained cells (0–100) by the staining intensity (0 to 3+, Supplementary Figure 1) (17).

### The Cancer Genome Atlas Database

The expression data of the KMO transcript, RNA-Seq by Expectation–Maximization (RSEM), was downloaded from the Broad GDAC Firehose data portal (https://gdac.broadinstitute.org/). KMO alterations and clinical data from patients with CRC were downloaded from the cbioportal (18, 19).

### Cell Culture

SW480, Caco-2, HT-29, HCT-116 HCT-15, and Lovo cell lines were obtained from the American Type Culture Collection (Manassas, VA). SW480, Caco-2, and Lovo cells were maintained in Dulbecco's Modified Eagle Medium (Gibco); HT-29, HCT116, and HCT15 cells were maintained in the RPMI 1640 medium. All culture media were supplemented with 10% FBS, 0.1-mM nonessential amino acids, 2-mM L-glutamine, and 100 U/mL penicillin–streptomycin. Cells were maintained in a 5% CO_2_ atmosphere at 37°C. The KMO inhibitor, UPF 648, was purchased from Axon Medchem (Reston, VA, USA) and dissolved in dimethyl sulfoxide (DMSO).

### Lentiviral Production and Infection

To knock down endogenous KMO, plasmids containing siRNA against KMO (siKMO) and vector were obtained from the National RNAi Core Facility Platform (Academia Sinica, Taiwan). The target sequence of KMO (5′-CCACAGGCTGTTGAAATGTAA-3′) located within the KMO CDS region was constructed into the vector. To make lentivirus, briefly, 293T cells were seeded and co-transfected with pCMVdR8.91, pMD.G, and siKMO, or control (siCtrl) plasmids using Lipofectamine 3000 (Thermo Fisher Scientific, Waltham, MA, USA) following the manufacturer's instructions. After 24–48 h of transfection, viral supernatants were harvested and stored at −80°C. Cells were infected in medium containing 8 μg/mL polybrene with lentivirus expressing siKMO or siCtrl for 24 h.

### Western Blot Analysis

Cells were trypsinized and harvested for further protein extraction; cell lysates were separated using SDS-PAGE electrophoresis as described previously (20). Antibodies against KMO (Abcam, Cambridge, MA, USA), Nanog, CD44, and β-actin (Cell Signaling, Danvers, MA, USA) were used. Protein levels were quantified using ImageJ software.

### Migration and Invasion Assays

As described previously (21), the migration and invasion assays were performed in 24-well-plates. SW480 (1 × 10^5^), Caco-2 (1.5 × 10^5^), or HT-29 cells (2 × 10^5^) in 200 μL of serum-free medium were seeded onto apical transwells with 8-μm pores (Greiner Bio One, Kremsmünster, Austria) or Matrigel matrix-coated transwell for migration and invasion assays, respectively. Complete medium (900 μL) was added to the lower chamber and incubated for 20 h. After incubation, the migrated or invaded cells were fixed with 100% methanol for 10 min and stained with 0.05% crystal violet for 1 h.

### Sphere Assay

Cells (5 × 10^2^) were seeded onto ultra-low attachment 96-well-plates (Corning, New York, NY, USA) and suspended in DMEM-F12 medium containing B-27 supplement, N2 supplement, recombinant human EGF, and recombinant human FGFβ (Gibco). After 7 days, tumorspheres were counted under a microscope.

### Cell Viability Assay

CRC cells (3 × 10^3^) were cultured in a 96-well-plate for 24 h and further treated with UPF 648 at concentrations indicated for 72 h. Cell viability was determined by colorimetric assay using 3-(4,5-dimethylthiazol-2-yl)-2,5-diphenyltetrazolium bromide (MTT) assay. Ten microliters of MTT solution (0.5 mg/mL, Sigma-Aldrich, St. Louis, MO, USA) was added to the medium and incubated at 37°C for 3 h. The violet precipitates were dissolved in 100 μL of dimethyl sulfoxide (DMSO), and the absorbance was measured at 570 nm using a UQuant spectrophotometer (BioTek Instruments, Winooski, VT, USA).

### OCR and ECAR Analyses

Cells (3 10^4^) were seeded into 24-well-plates for oxygen consumption rate (OCR) and extracellular acidification rate (ECAR) determination using a Seahorse Extracellular Flux XF-24 analyzer (Seahorse Bioscience, North Billerica, MA, USA) according to the manufacturer's instructions. Prior to the assay, the sensor cartridge was hydrated at 37°C in a non-CO_2_ incubator overnight. The culture medium was replaced with DMEM (pH 7.4) without sodium bicarbonate. Cells were incubated at 37°C in a non-CO_2_ incubator for 1 h. OCR and ECAR were determined before and after the injection of oligomycin (2 μg/mL), FCCP (5 μM), and antimycin A (5 μM). The OCR and ECAR values were analyzed using an XF-24 analyzer and normalized to cell number.

### Statistical Analysis

All calculations were performed using SPSS for Windows software, version 22 (SPSS, Chicago, IL, USA). A receiver operating characteristic curve (ROC) analysis was used to select the optimal cutoff values of KMO expression, including proteins (KMO H-score, Supplementary Figures 2A,B) and transcripts (KMO RSEM Supplementary Figures 2C,D), for defining low vs. high expression of KMO. The KMO expression level as test variable and patient's survival status as state variable were used to calculate coordinates of the ROC curve, sensitivity, and 1-specificity using SPSS software. The Youden index (22), the maximum value of sensitivity+specificity-1, was selected the optimal cutoff values. For survival analysis, overall survival (OS) and disease-free survival (DFS) curves of patients with CRC were plotted using the Kaplan–Meier method and compared using the log-rank test. The association between KMO expression and clinicopathological parameters was analyzed using contingency tables and the chi-square test. Statistical comparisons were performed using non-parametric tests, and statistical significance was defined as a *P* < 0.05.

## Results

### KMO Is Upregulated in CRC Tumor Tissues and Correlates With Poor Outcome

**Figure 1:**
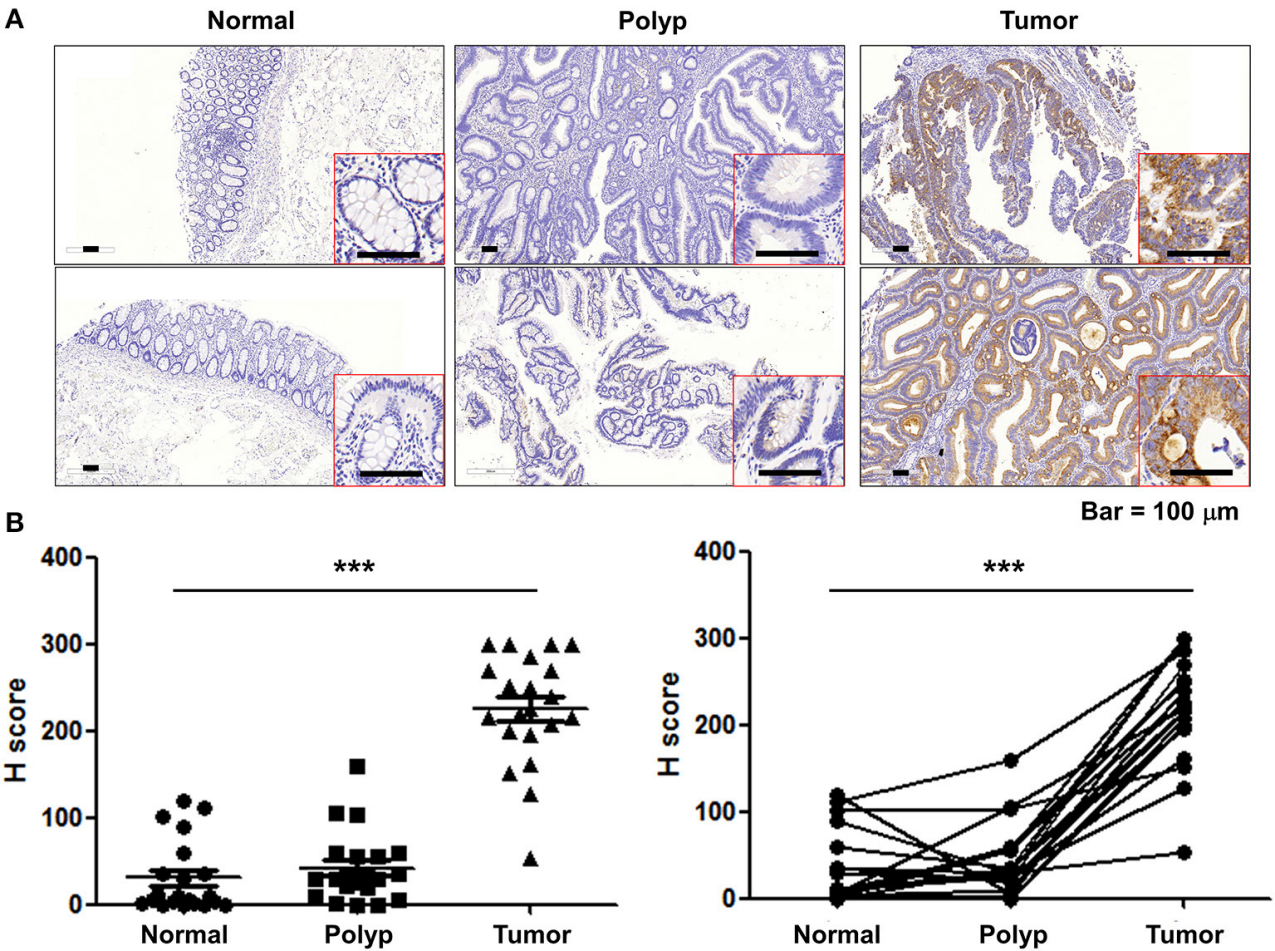
KMO are upregulated in CRC tumor tissues. **(A)** Representative images for KMO expressions in CRC tumor, paired polyps, and paired normal counterparts' specimens were detected by immunohistochemistry. Scar bar, 100 μm. **(B)** Scatter dot plots for H-score of KMO staining (*N* = 21). Student's *t*-test, ^***^P < 0.001.

**Figure 2:**
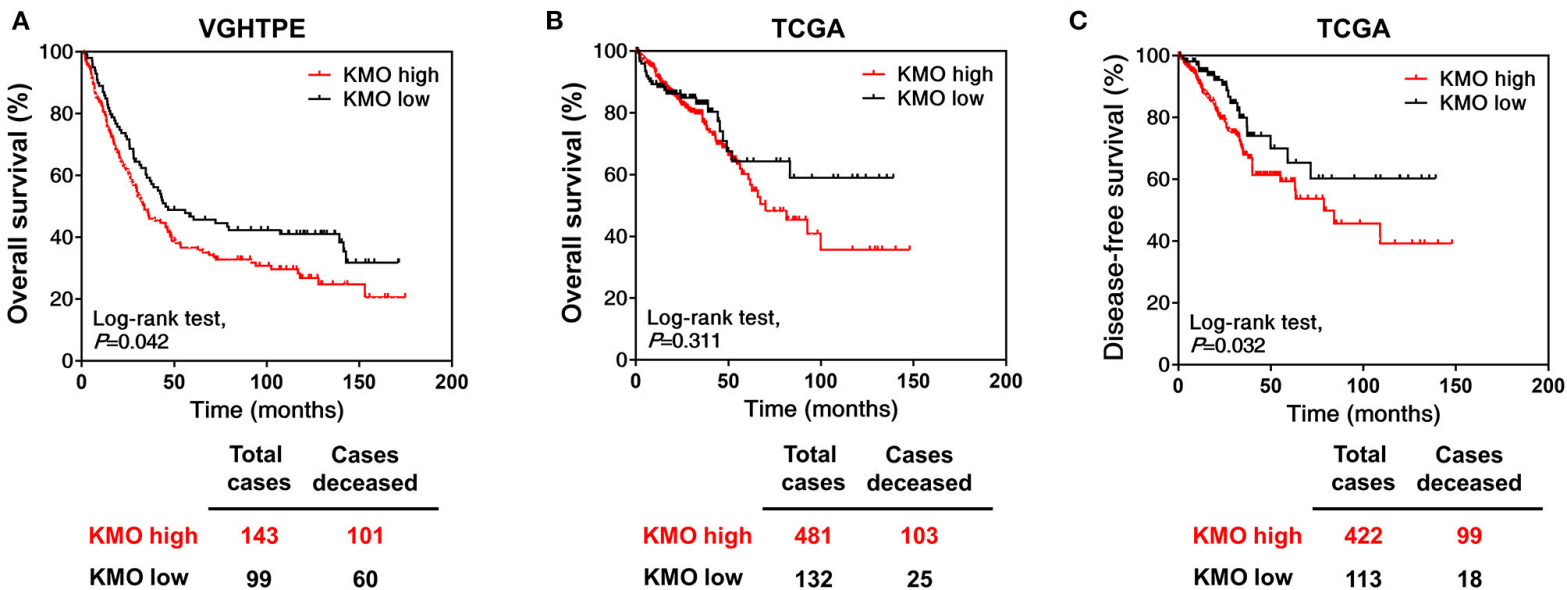
High KMO expression is associated with poor overall survival. **(A)** Overall survival of CRC patients from VGHTPE was plotted against time in month for the protein levels of KMO. **(B,C)** The level 3 data of mRNA RSEM from patients with CRC were selected from the TCGA and Broad GDAC Firehose data portal. Overall **(B)** and disease-free survival **(C)** curves were plotted for CRC patients with high or low transcript expressions of KMO.

To investigate the role of KMO in CRC tumorigenesis, we first examined the expression of KMO in tumors, paired polyps, and paired normal tissues by immunohistochemical staining (Figure 1A). The results showed that KMO expression in tumor tissues was significantly higher than that in normal tissues (Figure 1B). We further analyzed the association between clinicopathological characteristics and KMO expression in CRC patients from the VGHTPE cohort. KMO expression correlated with tumor metastasis but not age, sex, tumor location, pathology, AJCC stage, grade, or lymphovascular invasion (Table 1). Moreover, CRC patients with high KMO protein levels in the VGHTPE cohort had shorter survival (Figure 2A). To further validate the clinical relevance of KMO, we examined data from the TCGA database and found that CRC patients with higher KMO transcript levels were associated with worse DFS (Figures 2B,C). Higher KMO transcripts showed a decreasing trend in 5-year DFS (Supplementary Figure 3A). In addition, KMO gene alterations, including copy number variation, mutation, and mRNA dysregulation, correlated with poor OS (Supplementary Figure 3B). These results suggest that KMO might serve as a potential biomarker of CRC.

**Table 1:** Relationship of KMO expression with CRC clinicopathological parameters.

**Characteristics**	**KMO expression**		***P-*value**
	**Low (*n* = 99)**	**High (*n* = 143)**	
**Age**			
≤ 60	35 (35.4)	42 (29.4)	0.330
>60	64 (64.6)	101 (70.6)	
**Gender**			
Female	32 (32.3)	52 (36.4)	0.583
Male	67 (67.7)	91 (63.6)	
**Location**			
Left	55 (55.6)	87 (60.8)	0.428
Right	44 (44.4)	56 (39.2)	
**Pathology**			
Adenocarcinoma	92 (92.9)	139 (97.2)	0.194
Carcinoma	0 (0.0)	1 (0.7)	
Mucinous adenocarcinoma	6 (6.1)	3 (2.1)	
Signet ring cell carcinoma	1 (1.0)	0 (0.0)	
**AJCC**			
I	6 (6.1)	11 (7.7)	0.107
II	34 (34.3)	33 (23.1)	
III	32 (32.3)	41 (28.7)	
IV	27 (27.3)	58 (40.6)	
**High grade**			
No	90 (90.9)	125 (87.4)	0.495
Yes	7 (7.1)	14 (9.8)	
NA	2 (2.0)	4 (2.8)	
**Metastasis**			
No (AJCC I–III)	72 (72.7)	85 (59.4)	0.040
Yes (AJCC IV)	27 (27.3)	58 (40.6)	
**Lymphovascular invasion**			
No	76 (76.8)	98 (68.5)	0.115
Yes	17 (17.2)	38 (26.6)	
NA	6 (6.0)	7 (4.9)	
**MSI status**			
MSS	90 (90.9)	136 (95.1)	0.292
MSI-H	9 (9.1)	7 (4.9)	

*The cutoff value of the H-score of KMO was selected as 184.5*.

*AJCC, American Joint Commission on Cancer; CRC, colorectal cancer; KMO, kynurenine 3-monooxygenase; MSI, microsatellite instable; MSS, microsatellite stable; MSI-H, microsatellite instable-high; NA, not available*.

### Knockdown KMO Represses Sphere Formation, Migration, and Invasion Abilities of CRC Cells

**Figure 3:**
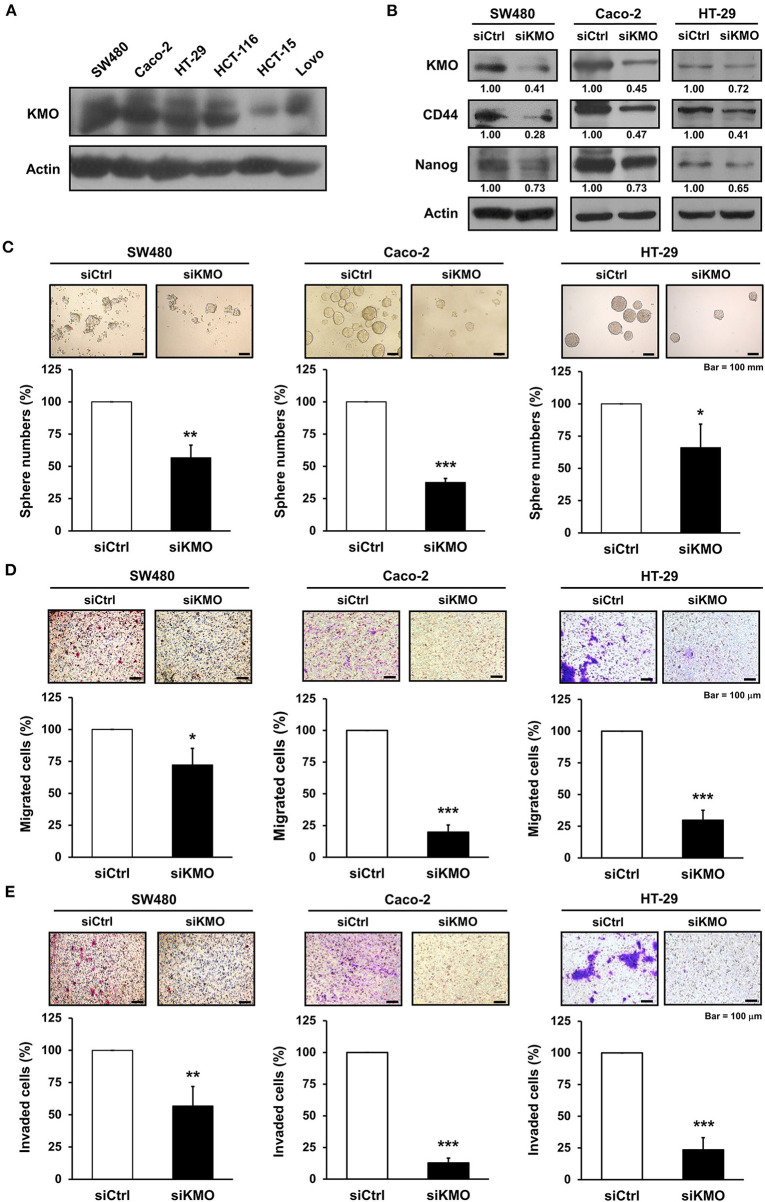
Knockdown of KMO suppresses stemness properties and motility of CRC cells. **(A)** Whole-cell extracts of SW480, Caco-2, HT-29, HCT-116, HCT-15, and Lovo cells were analyzed by western blot analysis using anti-KMO and anti-β-actin antibodies. **(B–E)** Whole-cell extracts of SW480, Caco-2, and HT-29 cells transduced with virus containing KMO siRNA (siKMO) or control (siCtrl) were harvested for western blot analysis using anti-KMO, anti-CD44, anti-Nanog, and anti-β-actin antibodies **(B)**, sphere **(C)**, transwell migration **(D)**, and invasion assays **(E)**. Means ± SD of three independent experiments performed in triplicate are shown [100 magnification times for **(D,E)**]. Student's *t*-test, ^*^P < 0.05; ^**^P < 0.01; ^***^P < 0.001.

We found that KMO was expressed in human CRC cell lines including SW480, Caco-2, HT-29, HCT-116, HCT-15, and Lovo cells (Figure 3A). To elucidate the function of KMO in CRC carcinogenesis, CRC cells were transfected with plasmids containing siRNA against KMO or control vector. Immunoblotting data showed that the expression of stemness markers, including CD44 and Nanog, was decreased in KMO-knockdown cells (Figure 3B). Sphere formation was significantly suppressed by KMO knockdown (Figure 3C). In addition, cell migration and invasion were also repressed in KMO-knockdown cells relative to control cells (Figures 3D,E). These data suggest that KMO promotes cancer progression in human CRC cells.

### KMO Inhibition Suppresses Cell Motility and Sphere Formation in CRC Cells

**Figure 4:**
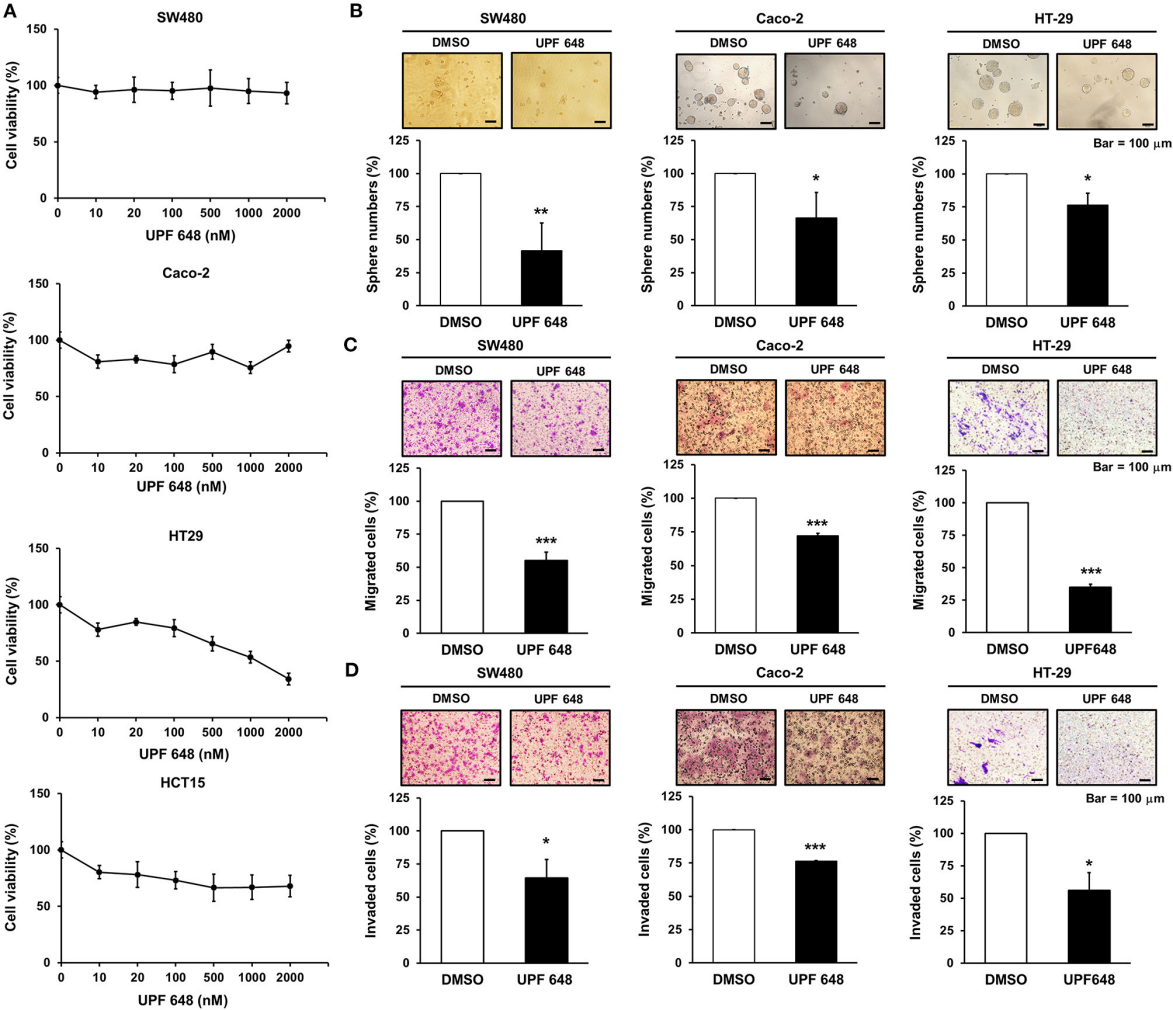
KMO inhibitor reduces the abilities of sphere formation, migration, and invasion in CRC cells. **(A)** SW480, Caco-2, HT-29, and HCT-15 cells were treated with indicated doses of UPF 648 for 72 h and examined by MTT assay. **(B–D)** SW480, Caco-2, and HT-29 cells treated with UPF 648 (1 μM) or DMSO were seeded for sphere **(B)**, transwell migration **(C)**, and invasion assays **(D)**. Means ± SD of three independent experiments performed in triplicate are shown [100 magnification times for **(C,D)**]. Student's *t*-test, ^*^P < 0.05; ^**^P < 0.01; ^***^P < 0.001.

KMO is an outer mitochondrial membrane enzyme that controls kynurenine catabolism. KMO inhibitors, which suppress KMO activity (16), were used to address the role of KMO activity in CRC progression. Inhibition of KMO with UPF 648 did not affect the OCR or basal ECAR of CRC cells (Supplementary Figure 4). UPF 648 exerted differential effects on the viability of CRC cells (Figure 4A). Nevertheless, the number of spheres, cell migration, and invasion were diminished by UPF 648 treatment in SW480, HT-29, and Caco-2 cells (Figures 4B–D). Likewise, sphere number and cell motility were reduced by the other well-explored KMO inhibitor, Ro 61–8048 (Supplementary Figures 5A–C). Taken together, these findings suggest the clinical significance and oncogenic role of KMO in CRC.

## Discussion

CRC still has a poor prognosis due to the high frequency of metastasis, recurrence, and drug resistance. As a result, identifying the prognostic factors and developing novel therapeutic strategies for CRC treatment is important. Inflammation is one of the hallmarks of cancer, and pro-inflammatory conditions enhance CRC progression and metastasis (23). KMO is upregulated by pro-inflammatory cytokines (24, 25). In the current study, we observed dysregulation of KMO in CRC. KMO expression was higher in CRC tumor tissues than in healthy tissues and polyps (Figure 1). In addition, high levels of KMO in patients with CRC correlated with worse survival rates (Figure 2).

Cancer stem cells (CSCs) are defined as tumor-initiating cells with the ability to self-renew and differentiate. Increasing evidence suggests that CSCs participate in tumor growth, metastasis, and recurrence (26). Targeting CSC is considered an effective anti-tumor strategy, including in CRC (27). CD44 is a surface marker of colorectal CSCs (28). CD44 knockdown suppresses clonal formation and tumorigenesis of CRC *in vivo* (29). The transcription factor Nanog regulates pluripotent genes and EMT (30, 31). It has been reported that high expression of Nanog is associated with poor prognosis and lymph node metastasis in CRC (32). Data revealed that knockdown of KMO decreased the number of CRC spheres with a reduction in CSC markers, including Nanog and CD44. Migration and invasion abilities were also reduced by KMO knockdown (Figure 3). Although our finding suggests a possible link of KMO to cancer stemness, consolidative supporting evidence is required. Further research is needed to validate the role of KMO in CRC stemness and to elucidate the underlying mechanisms of KMO in the regulation of expressions of CSC markers in CRC.

Miscellaneous KMO inhibitors have been developed and investigated for neurodegenerative disorders (33), for instance, a KMO tight-binding inhibitor, UPF 648, which is able to cross the blood–brain barrier in targeted therapies against neurodegenerative diseases (34). UPF648 treatment significantly reduces 3-HK levels in the brain and exerts neuroprotection (35). Ro 61–8048 and mNBA, both KMO inhibitors, exert neuroprotective effects by reducing 3-HK and quinolinic acid levels (36). Our results manifested that inhibition of KMO activity represses cell migration, invasion, and tumor sphere formation (Figure 4, Supplementary Figure 4). Notably, we observed that KMO inhibition showed different effects on cell viability and sphere formation. Previous studies indicate that inhibition of druggable genes shows diverse phenotypic outcomes between 2D monolayer culture and 3D sphere formation assays. The microenvironment affects phenotypic responses, suggesting that multiplexed assays render comprehensive information on anticancer target screening (37). Our results highlight the feasibility of KMO inhibitors in CRC treatment.

Recently, immunomodulation has increasingly played a key role in treating metastatic CRC. The tumor microenvironment comprises host stromal cells, tumor cells, and immune cells, including macrophages and leukocytes. Growing evidence suggests that the tumor microenvironment plays a crucial role in tumor progression and may serve as a therapeutic target (38). Targeting amino acid-metabolizing enzymes, which are involved in the regulation of immunosuppression, is a potential strategy for cancer treatment. It is well-known that L-tryptophan metabolism via the kynurenine pathway is involved in immune regulation. In the kynurenine pathway, IDO is the main rate-limiting enzyme and KMO is the downstream enzyme of IDO. In addition, IDO1 enhances T regulatory cell differentiation and further leads to immunosuppressive myeloid-derived suppressor cell recruitment (39, 40). IDO may serve as a predictive marker of distant metastasis in the early stages of CRC (41). High expression of IDO promotes tryptophan catabolite production, leading to immune escape and defeat of T cell invasion and contributing to CRC progression (42). On the other hand, previous studies indicated that IDO inhibitor 1-L-MT suppressed colitis-associated CRC through cell cycle arrest in an adaptive immunity modulation-independent manner (43). KMO is broadly expressed in various cell types, including immune cells such as macrophages, monocytes, and microglia (44). Nevertheless, the biological function of KMO, the downstream enzyme of IDO, on immunomodulation in CRC is still unclear. The interaction between KMO, IDO, and immunomodulation remains an unmet need.

Our study has some limitations; first, the interpretation of immunohistochemical staining and H-score determination may have interpersonal variations and future machine learning-based digital pathology should help to reduce the errors and variations. Secondly, despite that the current study showed that KMO inhibition by inhibitors possessed some anticancer effects on CRC cells, our study did not examine the effects of KMO inhibition on 3-HK production and the subsequent possible biological impact on CRC cells. The mechanisms of the anticancer effects of KMO inhibition in CRC cells require further investigation.

In summary, our results demonstrated that KMO is upregulated in CRC tissues and linked to worse survival. Inhibition of KMO reduces CRC progression *in vitro*. Our study suggests that KMO may act as an oncogene and reveals the therapeutic potential of targeting KMO enzymatic activity in CRC.

## Data Availability Statement

The original contributions presented in the study are included in the article/Supplementary Material, further inquiries can be directed to the corresponding author/s.

## Ethics Statement

The studies involving human participants were reviewed and approved by Institutional Review Board of Taipei Veterans General Hospital. The medical residual samples were acquired from the residual sample bank of Taipei Veterans General Hospital. VGHIRB waived the requirement for the use of an informed consent form.

## Author Contributions

C-YL and H-WT: conceptualization, supervision, and validation. T-TH, J-LC, and P-YC: data curation. C-HL, J-LC, Y-HL, and Y-YC: investigation. H-CL, S-HY, J-KJ, W-SC, and YC: methodology. C-YL, T-TH, J-LC, C-HL, and H-WT: writing—original draft. All authors had substantial contributions to the conception or design of the work, read the final manuscript, and agreed with the accuracy integrity of all parts of the work.

## Conflict of Interest

The authors declare that the research was conducted in the absence of any commercial or financial relationships that could be construed as a potential conflict of interest.
