# Supplementary material for: Significance of Kynurenine 3-Monooxygenase Expression in Colorectal Cancer
Source: Front Oncol. 2021 Apr 16;11:620361. doi: 10.3389/fonc.2021.620361 (PMC8085544; doi:10.3389/fonc.2021.620361)
Supplement: Supplementary file 1 [file Data_Sheet_1.docx]

**Supplementary material**

**
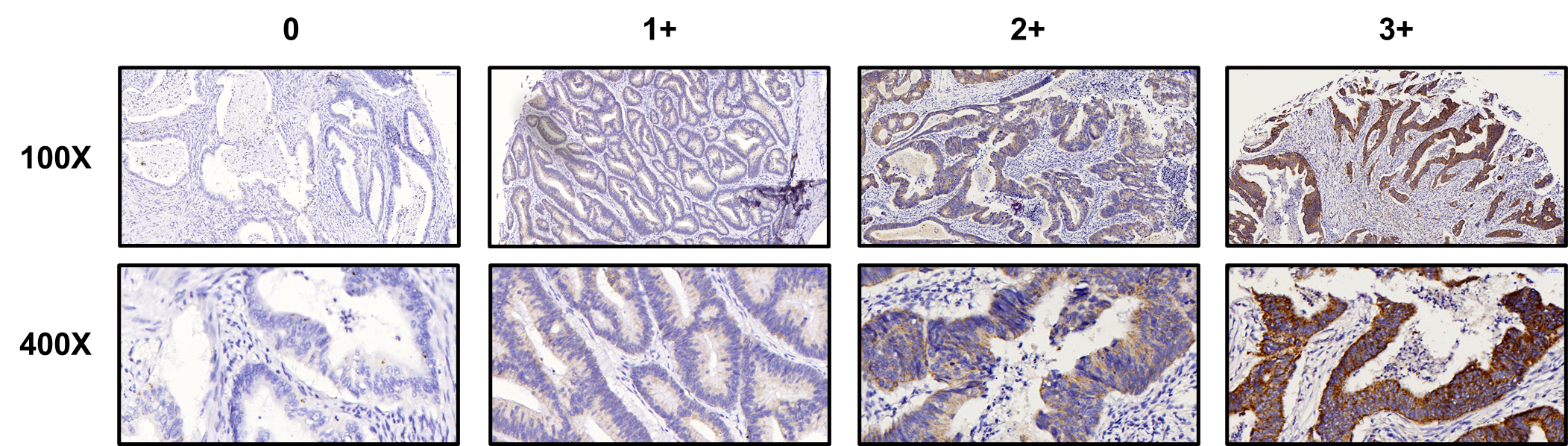
**

**Figure S1.** **Representative images of immunohistochemical staining of KMO in CRC tissues.**

The staining intensity (0, 1+, 2+ and 3+) was represented.

**
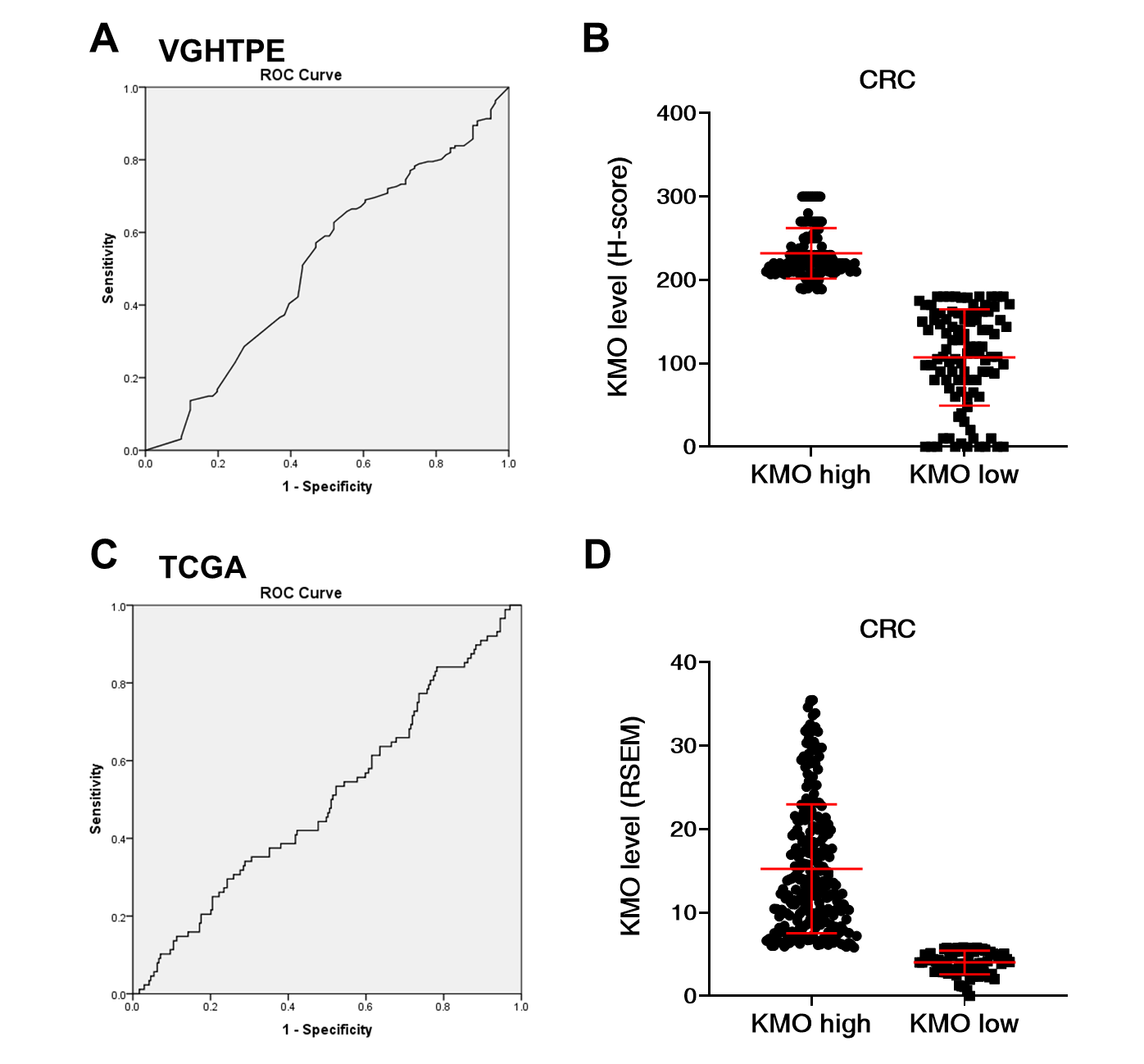
**

**Figure S2.** **ROC curves and histograms of dichotomization.**

(A, B) ROC curve analysis to select the optimal cut-off values of KMO expression in discriminating KMO high and KMO low from VGHTPE samples. (C, D) ROC curve analysis to select the optimal cut-off values of KMO expression in discriminating KMO high and KMO low from TCGA database.

**
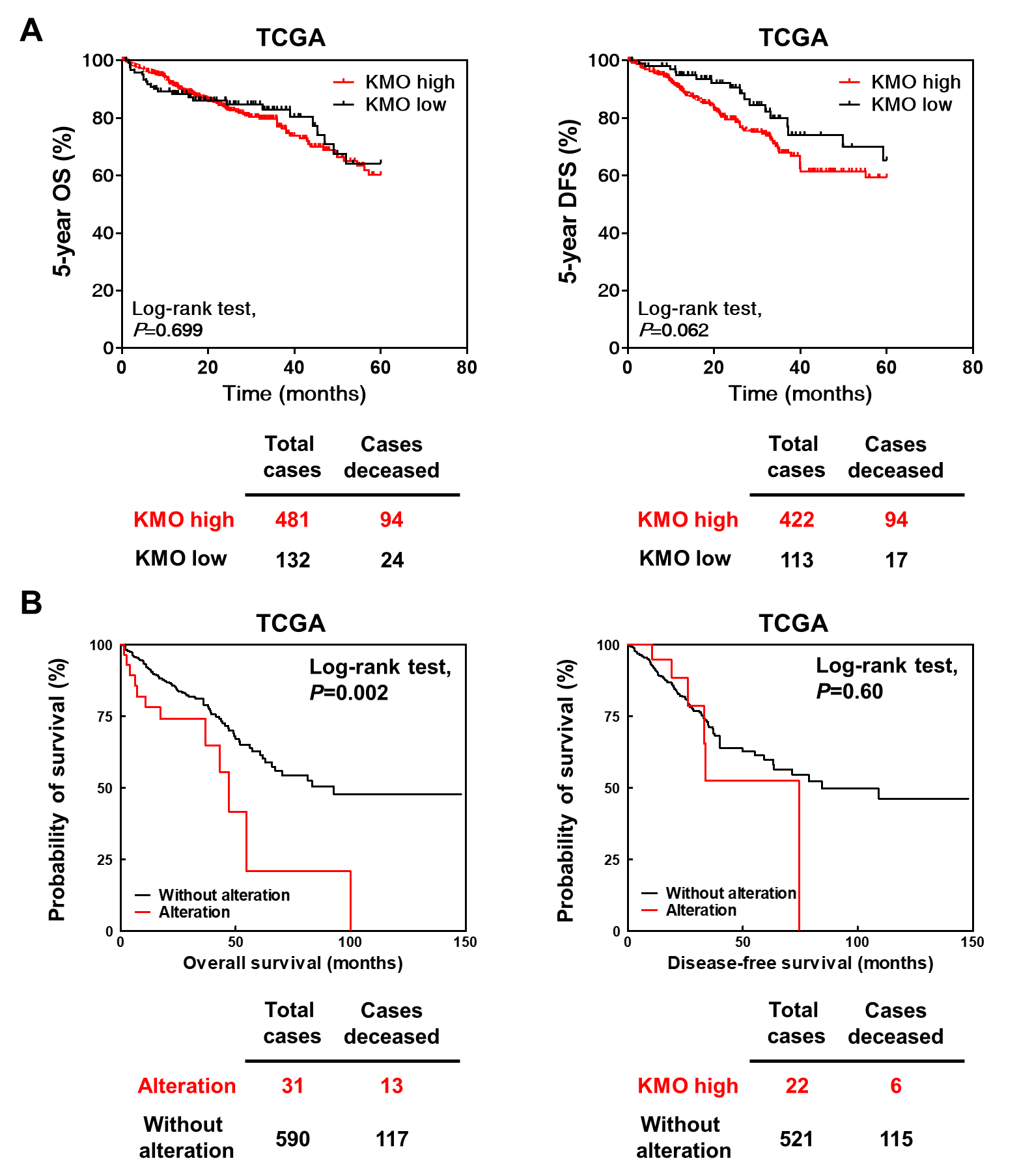
**

**Figure S3. KMO gene alterations relate to poor overall survival.**

(A, B) 5-year OS (A) and DFS (B) of CRC patients from TCGA database was plotted against time in month for the protein levels of KMO. (C, D) Data from patients with CRC were selected from the TCGA and Broad GDAC Firehose data portal. OS (C) and DFS (D) curves were plotted for CRC cases with or without alteration of KMO.


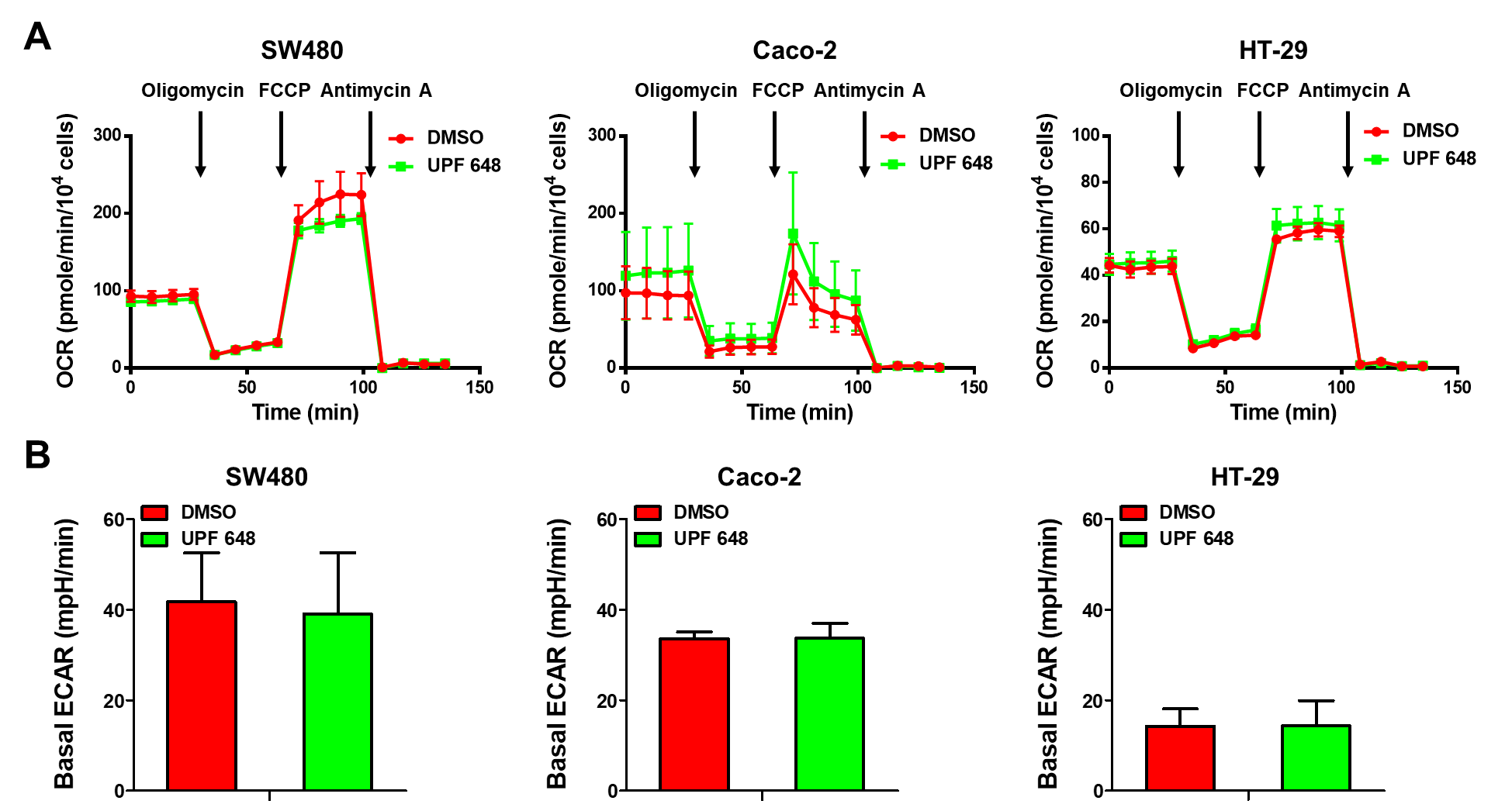


**Figure S4. KMO inhibitor did not alter mitochondrial functions of CRC cells.**

(A, B) SW480, Caco-2 and HT-29 cells treated with UPF 648 or DMSO for 48 h were determined the oxygen consumption rate (OCR) and extracellular acidification rate (ECAR).


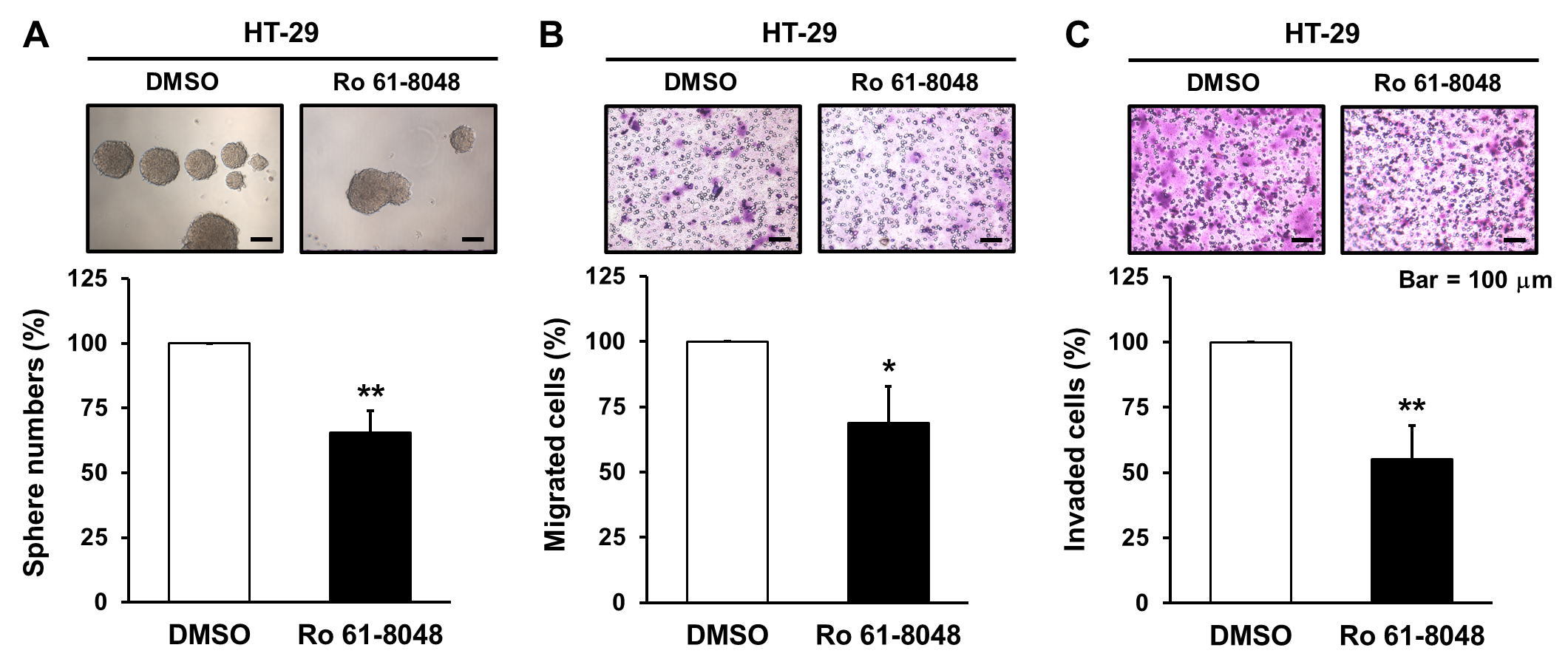


**Figure S5. KMO inhibitor suppresses sphere formation and cell motility of CRC cells.**

(A-C) HT-29 cells treated with Ro 61-8048 (1 μM) or DMSO were examined by sphere (A), migration (B) and invasion (C) assays.
